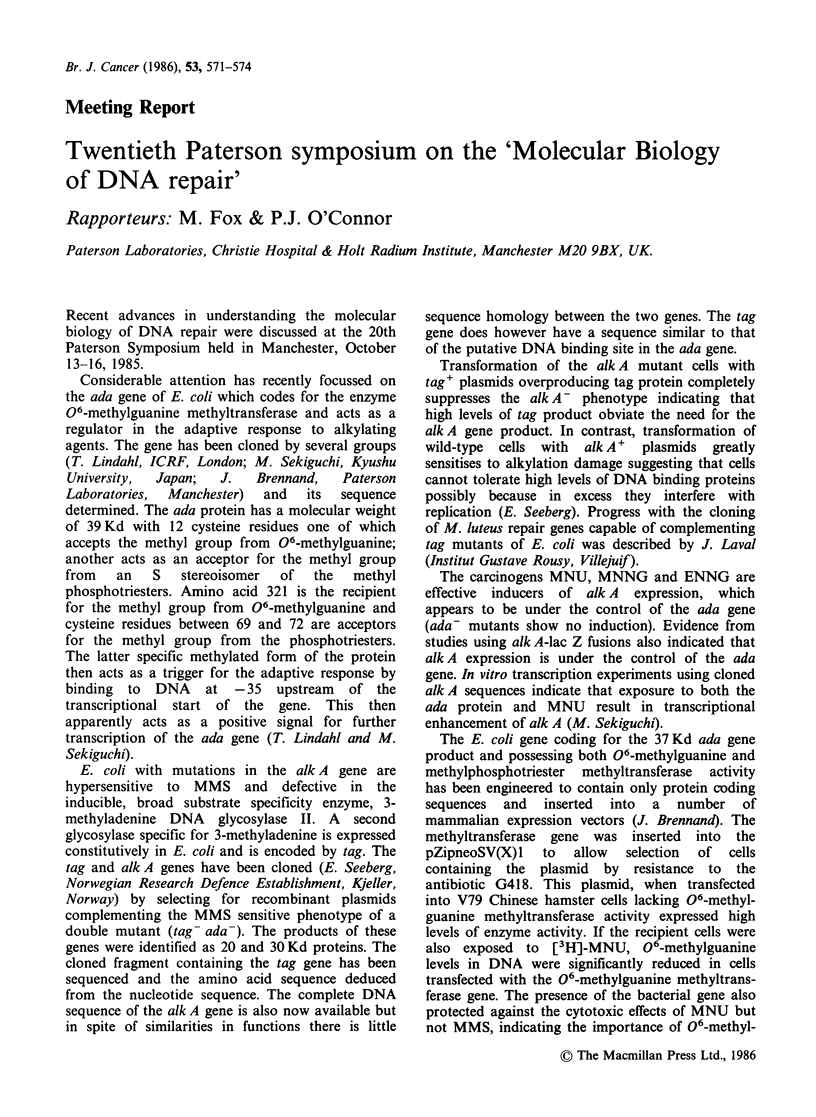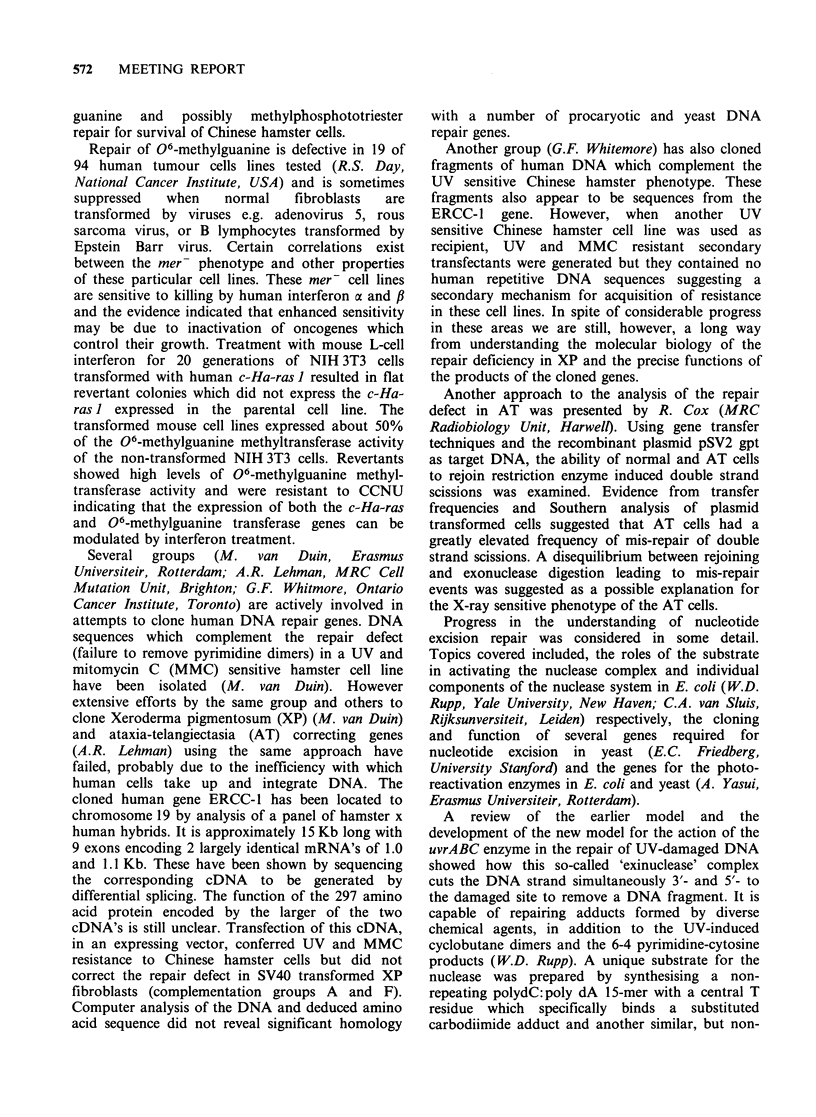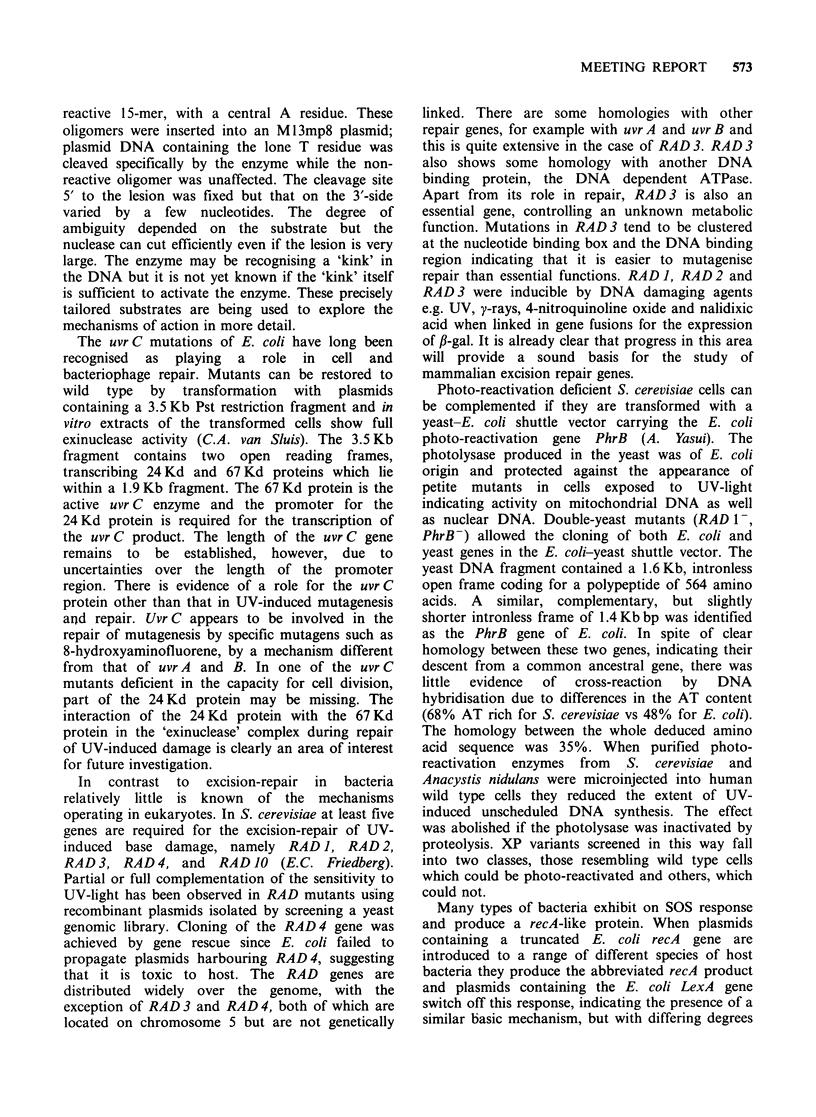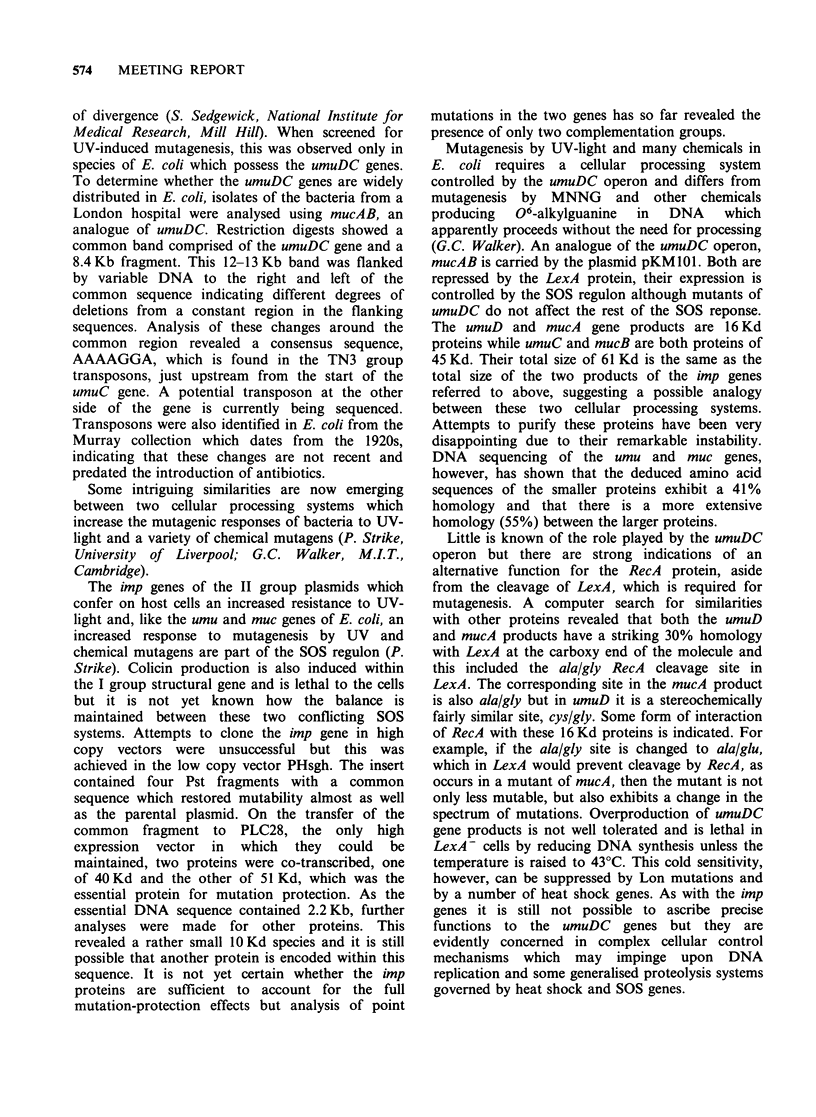# Twentieth Paterson symposium on `Molecular Biology of DNA repair'

**Published:** 1986-04

**Authors:** 


*Br. J. Cancer* (1986), **53**, 571–574

### Meeting Report

### Twentieth Paterson symposium on the 'Molecular Biology of DNA repair'

*Rapporteurs:* M. Fox & P.J. O'Connor

*Paterson Laboratories, Christie Hospital & Holt Radium Institute, Manchester M20 9BX, UK.*

Recent advances in understanding the molecular biology of DNA repair were discussed at the 20th Paterson Symposium held in Manchester, October 13–16, 1985.

Considerable attention has recently focussed on the *ada* gene of *E. coli* which codes for the enzyme $O^6$-methylguanine methyltransferase and acts as a regulator in the adaptive response to alkylating agents. The gene has been cloned by several groups (*T. Lindahl, ICRF, London*; *M. Sekiguchi, Kyushu University, Japan*; *J. Brennand, Paterson Laboratories, Manchester*) and its sequence determined. The *ada* protein has a molecular weight of 39 Kd with 12 cysteine residues one of which accepts the methyl group from $O^6$-methylguanine; another acts as an acceptor for the methyl group from an S stereoisomer of the methyl phosphotriesters. Amino acid 321 is the recipient for the methyl group from $O^6$-methylguanine and cysteine residues between 69 and 72 are acceptors for the methyl group from the phosphotriesters. The latter specific methylated form of the protein then acts as a trigger for the adaptive response by binding to DNA at −35 upstream of the transcriptional start of the gene. This then apparently acts as a positive signal for further transcription of the *ada* gene (*T. Lindahl and M. Sekiguchi*).

*E. coli* with mutations in the *alk A* gene are hypersensitive to MMS and defective in the inducible, broad substrate specificity enzyme, 3-methyladenine DNA glycosylase II. A second glycosylase specific for 3-methyladenine is expressed constitutively in *E. coli* and is encoded by *tag*. The *tag* and *alk A* genes have been cloned (*E. Seeberg, Norwegian Research Defence Establishment, Kjeller, Norway*) by selecting for recombinant plasmids complementing the MMS sensitive phenotype of a double mutant (*tag*$^-$ *ada*$^-$). The products of these genes were identified as 20 and 30 Kd proteins. The cloned fragment containing the *tag* gene has been sequenced and the amino acid sequence deduced from the nucleotide sequence. The complete DNA sequence of the *alk A* gene is also now available but in spite of similarities in functions there is little sequence homology between the two genes. The *tag* gene does however have a sequence similar to that of the putative DNA binding site in the *ada* gene.

Transformation of the *alk A* mutant cells with *tag*$^+$ plasmids overproducing tag protein completely suppresses the *alk A*$^-$ phenotype indicating that high levels of *tag* product obviate the need for the *alk A* gene product. In contrast, transformation of wild-type cells with *alk A*$^+$ plasmids greatly sensitises to alkylation damage suggesting that cells cannot tolerate high levels of DNA binding proteins possibly because in excess they interfere with replication (*E. Seeberg*). Progress with the cloning of *M. luteus* repair genes capable of complementing *tag* mutants of *E. coli* was described by *J. Laval* (*Institut Gustave Rousy, Villejuif*).

The carcinogens MNU, MNNG and ENNG are effective inducers of *alk A* expression, which appears to be under the control of the *ada* gene (*ada*$^-$ mutants show no induction). Evidence from studies using *alk A*-lac Z fusions also indicated that *alk A* expression is under the control of the *ada* gene. *In vitro* transcription experiments using cloned *alk A* sequences indicate that exposure to both the *ada* protein and MNU result in transcriptional enhancement of *alk A* (*M. Sekiguchi*).

The *E. coli* gene coding for the 37 Kd *ada* gene product and possessing both $O^6$-methylguanine and methylphosphotriester methyltransferase activity has been engineered to contain only protein coding sequences and inserted into a number of mammalian expression vectors (*J. Brennand*). The methyltransferase gene was inserted into the pZipneoSV(X)1 to allow selection of cells containing the plasmid by resistance to the antibiotic G418. This plasmid, when transfected into V79 Chinese hamster cells lacking $O^6$-methylguanine methyltransferase activity expressed high levels of enzyme activity. If the recipient cells were also exposed to [$^3$H]-MNU, $O^6$-methylguanine levels in DNA were significantly reduced in cells transfected with the $O^6$-methylguanine methyltransferase gene. The presence of the bacterial gene also protected against the cytotoxic effects of MNU but not MMS, indicating the importance of $O^6$-methyl-

© The Macmillan Press Ltd., 1986

guanine and possibly methylphosphototriester repair for survival of Chinese hamster cells.

Repair of $O^6$-methylguanine is defective in 19 of 94 human tumour cells lines tested (*R.S. Day, National Cancer Institute, USA*) and is sometimes suppressed when normal fibroblasts are transformed by viruses e.g. adenovirus 5, rous sarcoma virus, or B lymphocytes transformed by Epstein Barr virus. Certain correlations exist between the *mer*$^-$ phenotype and other properties of these particular cell lines. These *mer*$^-$ cell lines are sensitive to killing by human interferon $\alpha$ and $\beta$ and the evidence indicated that enhanced sensitivity may be due to inactivation of oncogenes which control their growth. Treatment with mouse L-cell interferon for 20 generations of NIH 3T3 cells transformed with human *c-Ha-ras 1* resulted in flat revertant colonies which did not express the *c-Ha-ras 1* expressed in the parental cell line. The transformed mouse cell lines expressed about 50% of the $O^6$-methylguanine methyltransferase activity of the non-transformed NIH 3T3 cells. Revertants showed high levels of $O^6$-methylguanine methyltransferase activity and were resistant to CCNU indicating that the expression of both the *c-Ha-ras* and $O^6$-methylguanine transferase genes can be modulated by interferon treatment.

Several groups (*M. van Duin, Erasmus Universiteir, Rotterdam; A.R. Lehman, MRC Cell Mutation Unit, Brighton; G.F. Whitmore, Ontario Cancer Institute, Toronto*) are actively involved in attempts to clone human DNA repair genes. DNA sequences which complement the repair defect (failure to remove pyrimidine dimers) in a UV and mitomycin C (MMC) sensitive hamster cell line have been isolated (*M. van Duin*). However extensive efforts by the same group and others to clone Xeroderma pigmentosum (XP) (*M. van Duin*) and ataxia-telangiectasia (AT) correcting genes (*A.R. Lehman*) using the same approach have failed, probably due to the inefficiency with which human cells take up and integrate DNA. The cloned human gene ERCC-1 has been located to chromosome 19 by analysis of a panel of hamster x human hybrids. It is approximately 15 Kb long with 9 exons encoding 2 largely identical mRNA's of 1.0 and 1.1 Kb. These have been shown by sequencing the corresponding cDNA to be generated by differential splicing. The function of the 297 amino acid protein encoded by the larger of the two cDNA's is still unclear. Transfection of this cDNA, in an expressing vector, conferred UV and MMC resistance to Chinese hamster cells but did not correct the repair defect in SV40 transformed XP fibroblasts (complementation groups A and F). Computer analysis of the DNA and deduced amino acid sequence did not reveal significant homology with a number of procaryotic and yeast DNA repair genes.

Another group (*G.F. Whitemore*) has also cloned fragments of human DNA which complement the UV sensitive Chinese hamster phenotype. These fragments also appear to be sequences from the ERCC-1 gene. However, when another UV sensitive Chinese hamster cell line was used as recipient, UV and MMC resistant secondary transfectants were generated but they contained no human repetitive DNA sequences suggesting a secondary mechanism for acquisition of resistance in these cell lines. In spite of considerable progress in these areas we are still, however, a long way from understanding the molecular biology of the repair deficiency in XP and the precise functions of the products of the cloned genes.

Another approach to the analysis of the repair defect in AT was presented by *R. Cox* (*MRC Radiobiology Unit, Harwell*). Using gene transfer techniques and the recombinant plasmid pSV2 gpt as target DNA, the ability of normal and AT cells to rejoin restriction enzyme induced double strand scissions was examined. Evidence from transfer frequencies and Southern analysis of plasmid transformed cells suggested that AT cells had a greatly elevated frequency of mis-repair of double strand scissions. A disequilibrium between rejoining and exonuclease digestion leading to mis-repair events was suggested as a possible explanation for the X-ray sensitive phenotype of the AT cells.

Progress in the understanding of nucleotide excision repair was considered in some detail. Topics covered included, the roles of the substrate in activating the nuclease complex and individual components of the nuclease system in *E. coli* (*W.D. Rupp, Yale University, New Haven; C.A. van Sluis, Rijksunversiteit, Leiden*) respectively, the cloning and function of several genes required for nucleotide excision in yeast (*E.C. Friedberg, University Stanford*) and the genes for the photoreactivation enzymes in *E. coli* and yeast (*A. Yasui, Erasmus Universiteir, Rotterdam*).

A review of the earlier model and the development of the new model for the action of the *uvrABC* enzyme in the repair of UV-damaged DNA showed how this so-called 'exinuclease' complex cuts the DNA strand simultaneously 3'- and 5'- to the damaged site to remove a DNA fragment. It is capable of repairing adducts formed by diverse chemical agents, in addition to the UV-induced cyclobutane dimers and the 6-4 pyrimidine-cytosine products (*W.D. Rupp*). A unique substrate for the nuclease was prepared by synthesising a non-repeating polydC:poly dA 15-mer with a central T residue which specifically binds a substituted carbodiimide adduct and another similar, but nonreactive 15-mer, with a central A residue. These oligomers were inserted into an M13mp8 plasmid; plasmid DNA containing the lone T residue was cleaved specifically by the enzyme while the non-reactive oligomer was unaffected. The cleavage site 5′ to the lesion was fixed but that on the 3′-side varied by a few nucleotides. The degree of ambiguity depended on the substrate but the nuclease can cut efficiently even if the lesion is very large. The enzyme may be recognising a 'kink' in the DNA but it is not yet known if the 'kink' itself is sufficient to activate the enzyme. These precisely tailored substrates are being used to explore the mechanisms of action in more detail.

The *uvr C* mutations of *E. coli* have long been recognised as playing a role in cell and bacteriophage repair. Mutants can be restored to wild type by transformation with plasmids containing a 3.5 Kb Pst restriction fragment and *in vitro* extracts of the transformed cells show full exinuclease activity (*C.A. van Sluis*). The 3.5 Kb fragment contains two open reading frames, transcribing 24 Kd and 67 Kd proteins which lie within a 1.9 Kb fragment. The 67 Kd protein is the active *uvr C* enzyme and the promoter for the 24 Kd protein is required for the transcription of the *uvr C* product. The length of the *uvr C* gene remains to be established, however, due to uncertainties over the length of the promoter region. There is evidence of a role for the *uvr C* protein other than that in UV-induced mutagenesis and repair. *Uvr C* appears to be involved in the repair of mutagenesis by specific mutagens such as 8-hydroxyaminofluorene, by a mechanism different from that of *uvr A* and *B*. In one of the *uvr C* mutants deficient in the capacity for cell division, part of the 24 Kd protein may be missing. The interaction of the 24 Kd protein with the 67 Kd protein in the 'exinuclease' complex during repair of UV-induced damage is clearly an area of interest for future investigation.

In contrast to excision-repair in bacteria relatively little is known of the mechanisms operating in eukaryotes. In *S. cerevisiae* at least five genes are required for the excision-repair of UV-induced base damage, namely *RAD 1, RAD 2, RAD 3, RAD 4,* and *RAD 10* (*E.C. Friedberg*). Partial or full complementation of the sensitivity to UV-light has been observed in *RAD* mutants using recombinant plasmids isolated by screening a yeast genomic library. Cloning of the *RAD 4* gene was achieved by gene rescue since *E. coli* failed to propagate plasmids harbouring *RAD 4*, suggesting that it is toxic to host. The *RAD* genes are distributed widely over the genome, with the exception of *RAD 3* and *RAD 4*, both of which are located on chromosome 5 but are not genetically linked. There are some homologies with other repair genes, for example with *uvr A* and *uvr B* and this is quite extensive in the case of *RAD 3*. *RAD 3* also shows some homology with another DNA binding protein, the DNA dependent ATPase. Apart from its role in repair, *RAD 3* is also an essential gene, controlling an unknown metabolic function. Mutations in *RAD 3* tend to be clustered at the nucleotide binding box and the DNA binding region indicating that it is easier to mutagenise repair than essential functions. *RAD 1, RAD 2* and *RAD 3* were inducible by DNA damaging agents e.g. UV, $\gamma$-rays, 4-nitroquinoline oxide and nalidixic acid when linked in gene fusions for the expression of $\beta$-gal. It is already clear that progress in this area will provide a sound basis for the study of mammalian excision repair genes.

Photo-reactivation deficient *S. cerevisiae* cells can be complemented if they are transformed with a yeast–*E. coli* shuttle vector carrying the *E. coli* photo-reactivation gene *PhrB* (*A. Yasui*). The photolysase produced in the yeast was of *E. coli* origin and protected against the appearance of petite mutants in cells exposed to UV-light indicating activity on mitochondrial DNA as well as nuclear DNA. Double-yeast mutants (*RAD* $1^-$, *PhrB*$^-$) allowed the cloning of both *E. coli* and yeast genes in the *E. coli*–yeast shuttle vector. The yeast DNA fragment contained a 1.6 Kb, intronless open frame coding for a polypeptide of 564 amino acids. A similar, complementary, but slightly shorter intronless frame of 1.4 Kb bp was identified as the *PhrB* gene of *E. coli*. In spite of clear homology between these two genes, indicating their descent from a common ancestral gene, there was little evidence of cross-reaction by DNA hybridisation due to differences in the AT content (68% AT rich for *S. cerevisiae* vs 48% for *E. coli*). The homology between the whole deduced amino acid sequence was 35%. When purified photo-reactivation enzymes from *S. cerevisiae* and *Anacystis nidulans* were microinjected into human wild type cells they reduced the extent of UV-induced unscheduled DNA synthesis. The effect was abolished if the photolysase was inactivated by proteolysis. XP variants screened in this way fall into two classes, those resembling wild type cells which could be photo-reactivated and others, which could not.

Many types of bacteria exhibit on SOS response and produce a *recA*-like protein. When plasmids containing a truncated *E. coli recA* gene are introduced to a range of different species of host bacteria they produce the abbreviated *recA* product and plasmids containing the *E. coli LexA* gene switch off this response, indicating the presence of a similar basic mechanism, but with differing degrees of divergence (*S. Sedgewick, National Institute for Medical Research, Mill Hill*). When screened for UV-induced mutagenesis, this was observed only in species of *E. coli* which possess the *umuDC* genes. To determine whether the *umuDC* genes are widely distributed in *E. coli*, isolates of the bacteria from a London hospital were analysed using *mucAB*, an analogue of *umuDC*. Restriction digests showed a common band comprised of the *umuDC* gene and a 8.4 Kb fragment. This 12–13 Kb band was flanked by variable DNA to the right and left of the common sequence indicating different degrees of deletions from a constant region in the flanking sequences. Analysis of these changes around the common region revealed a consensus sequence, AAAAGGA, which is found in the TN3 group transposons, just upstream from the start of the *umuC* gene. A potential transposon at the other side of the gene is currently being sequenced. Transposons were also identified in *E. coli* from the Murray collection which dates from the 1920s, indicating that these changes are not recent and predated the introduction of antibiotics.

Some intriguing similarities are now emerging between two cellular processing systems which increase the mutagenic responses of bacteria to UV-light and a variety of chemical mutagens (*P. Strike, University of Liverpool; G.C. Walker, M.I.T., Cambridge*).

The *imp* genes of the II group plasmids which confer on host cells an increased resistance to UV-light and, like the *umu* and *muc* genes of *E. coli*, an increased response to mutagenesis by UV and chemical mutagens are part of the SOS regulon (*P. Strike*). Colicin production is also induced within the I group structural gene and is lethal to the cells but it is not yet known how the balance is maintained between these two conflicting SOS systems. Attempts to clone the *imp* gene in high copy vectors were unsuccessful but this was achieved in the low copy vector PHsgh. The insert contained four Pst fragments with a common sequence which restored mutability almost as well as the parental plasmid. On the transfer of the common fragment to PLC28, the only high expression vector in which they could be maintained, two proteins were co-transcribed, one of 40 Kd and the other of 51 Kd, which was the essential protein for mutation protection. As the essential DNA sequence contained 2.2 Kb, further analyses were made for other proteins. This revealed a rather small 10 Kd species and it is still possible that another protein is encoded within this sequence. It is not yet certain whether the *imp* proteins are sufficient to account for the full mutation-protection effects but analysis of point mutations in the two genes has so far revealed the presence of only two complementation groups.

Mutagenesis by UV-light and many chemicals in *E. coli* requires a cellular processing system controlled by the *umuDC* operon and differs from mutagenesis by MNNG and other chemicals producing $O^6$-alkylguanine in DNA which apparently proceeds without the need for processing (*G.C. Walker*). An analogue of the *umuDC* operon, *mucAB* is carried by the plasmid pKM101. Both are repressed by the *LexA* protein, their expression is controlled by the SOS regulon although mutants of *umuDC* do not affect the rest of the SOS reponse. The *umuD* and *mucA* gene products are 16 Kd proteins while *umuC* and *mucB* are both proteins of 45 Kd. Their total size of 61 Kd is the same as the total size of the two products of the *imp* genes referred to above, suggesting a possible analogy between these two cellular processing systems. Attempts to purify these proteins have been very disappointing due to their remarkable instability. DNA sequencing of the *umu* and *muc* genes, however, has shown that the deduced amino acid sequences of the smaller proteins exhibit a 41% homology and that there is a more extensive homology (55%) between the larger proteins.

Little is known of the role played by the *umuDC* operon but there are strong indications of an alternative function for the *RecA* protein, aside from the cleavage of *LexA*, which is required for mutagenesis. A computer search for similarities with other proteins revealed that both the *umuD* and *mucA* products have a striking 30% homology with *LexA* at the carboxy end of the molecule and this included the *ala/gly RecA* cleavage site in *LexA*. The corresponding site in the *mucA* product is also *ala/gly* but in *umuD* it is a stereochemically fairly similar site, *cys/gly*. Some form of interaction of *RecA* with these 16 Kd proteins is indicated. For example, if the *ala/gly* site is changed to *ala/glu*, which in *LexA* would prevent cleavage by *RecA*, as occurs in a mutant of *mucA*, then the mutant is not only less mutable, but also exhibits a change in the spectrum of mutations. Overproduction of *umuDC* gene products is not well tolerated and is lethal in $LexA^-$ cells by reducing DNA synthesis unless the temperature is raised to 43°C. This cold sensitivity, however, can be suppressed by Lon mutations and by a number of heat shock genes. As with the *imp* genes it is still not possible to ascribe precise functions to the *umuDC* genes but they are evidently concerned in complex cellular control mechanisms which may impinge upon DNA replication and some generalised proteolysis systems governed by heat shock and SOS genes.